# Antibiotic properties of *Satureja montana* L. hydrolate in bacteria and fungus of clinical interest and its impact in non-target environmental microorganisms

**DOI:** 10.1038/s41598-022-22419-2

**Published:** 2022-11-02

**Authors:** María Rosa Pino-Otín, Cristina Gan, Eva Terrado, María Angeles Sanz, Diego Ballestero, Elisa Langa

**Affiliations:** 1 Universidad San Jorge, Campus Universitario Villanueva de Gállego Autovía A-23 Zaragoza-Huesca, Km. 510, Villanueva de Gállego, 50830 Zaragoza, Spain; 2 Universidad de Zaragoza, C. de Pedro Cerbuna, 12, 50009 Zaragoza, Spain; 3 CITA, Área de Laboratorios de Análisis y Asistencia Tecnológica, Avda. Montañana 930, 50059 Zaragoza, Spain

**Keywords:** Microbiology, Plant sciences, Natural hazards

## Abstract

The aim of this study was to analyse the microbicidal and microbiostatic activity of *S. montana* hydrolate L., the water-soluble fraction of the hydro-distillation process used to obtain the essential oil, on 14 Gram-positive and Gram-negative bacteria and a fungus of clinical interest. To consider whether this hydrolate is a more environmentally friendly alternative to traditional antibiotics, its effect on non-target microorganisms in the aquatic and terrestrial environment was analysed using natural soil and river microorganism communities, characterized through 16S rRNA gene sequencing. Results showed that *S. montana* hydrolate was especially effective (25% v/v concentration) against *Pasteurella aerogenes*, *Streptococcus agalactiae* and *Acinetobacter baumannii* (priority 1, WHO). It was also a microbicide for a further 7 bacterial strains and the fungus *Candida albicans* (50% v/v concentration). The river and soil communities exposed to the hydrolate showed a decrease in their growth, as well as a decrease in their ability to metabolize polymers and carbohydrates (soil microorganisms) and polymers, carboxylic and ketone acids (river microorganisms). Hydrolates could be an alternative to conventional antibiotics, but their impact on the environment must be taken into account.

## Introduction

Hydrolates are co-products from the distillation or hydro-distillation of aromatic plants to produce essential oils (EOs). Recently, hydrolates are become of interest in perfumery and cosmetics^1^, food flavouring^2^ and insect pest repellents^3^, and because of their herbicide^4^ and nematicide^5^ properties, among others.

In recent years, different hydrolates with antimicrobial activity have been described^6–9^, particularly to prevent the development of pathogenic and spoiling microorganisms in food products^2,10–12^.

Many essential oil producing and exporting countries, discard these hydrolates, which are therefore wasted. However, their growing applications can add value to this waste by lengthening its life cycle, in accordance with the principles of the circular economy. Hydrolates are cheap and easy to obtain^13^. In addition, their use as antibiotics can have advantages: they better maintain the organoleptic characteristics of food^10^, and they can be good alternatives to chlorine, avoiding the generation of harmful by-products that are derived from the reaction between chlorine and organic matter^11^. It is especially interesting that they may generate less resistance than synthetic antibiotics^14^. From a One health perspective, their impact on the environment is another aspect that should be elucidated.

The *Satureja* L. genus is reported within the family Lamiaceae^15^ and encompasses over 200 different herbs or shrub species, which are often aromatic, distributed in the Mediterranean area, Asia and some regions of America^16^. This genus shows notable pharmacological properties, such as antioxidant, anti-inflammatory or analgesic, among others^17–20^.

Hydrolates of different species of Satureja have also shown antimicrobial activity^10,21–24^, sometimes even higher than that presented by the essential oil (EO) from the same plant because they are active at lower concentrations. In addition, *Satureja hortensis* L. has antifungal properties^25^. To the best of our knowledge, the antimicrobial properties of *Satureja montana* hydrolate have only been studied in a few pathogenic bacteria^26^.

In a previous work, he most abundant volatile components of *Satureja montana* were identified as carvacrol, followed by thymol. Carvacrol and thymol are terpenoids with probed antimicrobial properties in bacteria^27–29^ and fungi^30^, suggesting that the hydrolate may show high bioactivity. However, for hydrolates or their constituents to be a good alternative to synthetic antibiotics, it is necessary for them to have less of an environmental impact or less microbial resistance than that of the treatments for many infection diseases today^31^. The WHO (2016)^32^ has stated that antibiotic resistance is one of the greatest threats to global health, food security and development. However, the demand for antibiotics, for both human use and for livestock^33^, continues to increase, and the high consumption of antibiotics causes elevated amounts of drugs and their metabolites to be released through wastewater^34^ and subsequently disseminated into the environment. Sewage treatment plants (STPs) are not usually able to remove these contaminants, so they can be found in effluent and sewage sludge, reaching rivers^35,36^ or soils when biosolids are used as fertilizers^37^.

The result is the occurrence of small concentrations of antibiotics in the environment, in the order of nanograms and micrograms per litre of wastewater or per kilogram of waste sludge^38–40^. This is the reason why they are usually known as “micropollutants”. Some antibiotics seem to reach higher levels in digested sludge (in the order of mg/kg), such as tetracycline in biosolids^41^. The effect of these micropollutants is not well understood, but there are numerous studies in the literature indicating that they can cause ecotoxicity in non-target aquatic organisms, such as cyanobacteria, algae, and some invertebrate and vertebrate species^42–44^, and in terrestrial organisms, such as crop plants^45,46^ and key terrestrial invertebrates, such as earthworms^47^. It is remarkable that antibiotics are still bioactive chemicals that are potentially hazardous to soil bacteria^45,48^ and also to aquatic microorganisms^42^ in the environment.

Despite the many properties described for hydrolates, including their antimicrobial activity, little is known about their potential impact on the environment or their ecotoxicity in non-target organisms. Pioneering studies of the ecotoxicity of *Lavandula luisieri* and *Artemisia absinthium* hydrolates on the aquatic^49^ and edaphic environments^50,51^, including soil and river microbial communities, show that hydrolates are not harmless and can affect non-target terrestrial and aquatic microorganisms.

The effect of *S. montana* hydrolate on communities of non-target microorganisms is currently unknown. However, it has been shown that hydrolate can produce environmental impact at low concentrations on individual non-target bioindicators in both water and soil^52^. The survival of the invertebrate *Daphnia magna* is affected by exposure to the hydrolate, as is that of the bacterium *Vibrio fisheri*. In the case of soil indicator organisms, *S. montana* hydrolate had a strong phytotoxic effect on the plant *Allium cepa* and on the survival of the earthworm *Eisenia fetida*, although the latter is the most resistant of the bioindicators studied. In addition, the river periphyton communities underwent changes in photosynthetic performance (LC_50_ = 4.23%), showing that *S. montana* hydrolate affects complex communities. The study of the effect of the hydrolate on water and soil microbial communities, the basis of many food chains and responsible for the maintenance of the carbon cycle, was still pending.

Therefore, the aim of this study was (1) to evaluate the antimicrobial properties of *S. montana* hydrolate in 15 microorganisms of clinical interest and compare its antimicrobial activity with that of its main volatile component (carvacrol); (2) to study the ecotoxicity of the hydrolate in non-target microbial river communities and non-target microbial communities of natural soil. The use of entire communities and not just individual organisms can help to generate a more complete overview of the impact of these products on soils and waters, from an environmental point of view.

## Material and methods

### Plant material and hydrolate

The hydrolate of *S. montana* L was kindly provided by the Center for Research and Agrofood Technology of Aragon (CITA) and obtained with the proper permissions from a population of 40 plants in Ejea de los Caballeros (Aragón, Spain, 42°8′8.73″ N, 1°12′31.50″ W/346 m a.s.l). The fresh biomass of these plants (75 kg) was distilled in a semi -industrial stainless-steel extraction pilot plant equipped with a pressure reducing valve as previously described^53^. The hydrolate (aqueous phase) was decanted from the essential oil in a separatory funnel and filtered before use (see Support information 3 for more details).

The volatile organic compound content of the hydrolate, previously analysed by gas chromatography-mass spectrometry, revealed the presence of one alcohol and six terpenoids, with carvacrol (89.03%) and thymol (6.66%) being the most abundant, and terpinen-4-ol (1.34%), 1-octen-3-ol (1.02%), borneol (0.99%), 1,8-cineole (0.56%) and linalool (0.41%) being found to a much lesser extent^52^.

### Solvents and reagents

Carvacrol (CAS: 499-75-2) with a minimum purity of 98.0% and a molecular weight of 150.22 g/mol was purchased from Sigma-Aldrich. The solvents used to dissolve carvacrol were absolute ethanol (CAS: 64-17-5) from Panreac Applichem, with a purity of 99.5%; dimethyl sulfoxide (DMSO) (CAS: 67-68-5) from Fisher Bioreagents, with a purity ≥ 99.7%, and polysorbate 80 (CAS: 9005-65-6) from Sigma-Aldrich, with a purity ≥ 99.8%. Distilled water was obtained from SIEMENS Ultra Clear™ Compact RO DI.

### Microorganisms

The following Gram-positive and Gram-negative reference bacterial strains were selected for this study: *Staphylococcus aureus* ATCC 9144, *Streptococcus agalactiae* ATCC 12386, *Enterococcus faecalis* ATCC 19433, *Bacillus subtilis* ATCC 6633, *Listeria monocytogenes* ATCC 7644, *Escherichia coli* ATCC 25922, *Salmonella typhimurium* ATCC 13311, *Klebsiella pneumoniae* C6, *Serratia marcescens* ATCC 13880, *Proteus mirabilis* ATCC 35659, *Pseudomonas aeruginosa* ATCC 27853, *Klebsiella aerogenes* ATCC 13048, *Acinetobacter baumannii* ATCC 19606 and *Pasteurella aerogenes* ATCC 27883. One fungus, *Candida albicans* ATCC 10231, was also included in this work.

All microorganisms were purchased from Thermo Scientific in the form of Culti-loops™ lyophilised bacteria/fungus and were revived (following the product sheet for every microorganism, see Support Information 1) and then stored at -80ºC in cryovials (obtained from Deltalab S.L. Barcelona, Spain) until use. Microorganism cultures for antimicrobial activity assays were grown according to the ATCC and Thermo Scientific product sheet for each strain (see Support Information 1).

The Gram-negative and Gram-positive bacterial strains used were aerobic. The bacteria types and the fungi were selected based on their clinical interest, as they currently cause some of the most common infections^54–58^, and because of their potential severity and their ability to generate resistance, according to the World Health Organisation's list of priority pathogens (WHO 2017).

### Determination of antibacterial activity

In order to study the antimicrobial properties of *S. montana* hydrolate and its main volatile compound, carvacrol, the minimum inhibitory concentrations (MICs) were obtained, using the broth microdilution method in round bottom 96-well plates, according to the Clinical and Laboratory Standards Institute guideline (SCLI, M07-A9 2018) and ISO 207776-1 (2019) for bacteria, and (SCLI, M27-A3, 2017) for *C. albicans*, as follows.

#### Carvacrol and hydrolate stock solution preparation

As carvacrol is a water-insoluble compound, ethanol, DMSO and polysorbate 80 were used to prepare the stock solutions for the subsequent antimicrobial tests.

The toxicity of the solvents on bacteria was examined by the microdilution method in a 96-well plate, obtaining the maximum innocuous concentration (MInC) to ensure that the concentrations used for the tests were completely harmless to the microorganisms. Carvacrol was dissolved depending on the bacterial strain used in each experiment, to achieve maximum solubility without affecting the bacteria. The table in Support Information 1 indicates which solvent was used to dissolve carvacrol in each test, depending on the type of bacteria. Prior to the antifungal and antibacterial analysis, all stock solutions were sonicated using an Ultrasons, J.P. SELECTA® analogue ultrasonic bath until a homogeneous and stable phase was obtained. The hydrolate (completely water soluble) was diluted directly in the corresponding culture broth.

#### 96-well plate preparation

For the tests, 100 µL of carvacrol stock solution or 100 µL of the hydrolate and 100 µL of culture broth were added to each well of the first column of the 96-well plate. Serial twofold dilutions were then applied from column 1 to 10, giving a final volume of 100 µL in each well and resulting in a concentration range between 4 and 2000 µg/mL for carvacrol and between 0.1 and 50% for the hydrolate. A positive control (wells with broth and inoculum but without testing product) and negative control (wells without inoculum and without testing product) were also included in each experiment, in columns 11 and 12, respectively. Bacteria were revived from the cryovials 24 h before the experiment, using the broth and conditions specified in Support Information1. Fifteen minutes before inoculating the plates, the bacterial cultures were adjusted to the McFarland standard (SLCI, 2018), by spectrophotometry at 625 nm wavelength, to achieve an approximate bacterial concentration of 5 × 10^8^ CFU/mL. The cultures were then diluted 1:20 with broth, and 10 µL were inoculated into each well, except in those designated as negative controls, to reach an approximate working concentration of 2.5 × 10^5^ CFU/mL.

The *C. albicans* inoculum was also adjusted to the 0.5 Mcfarland standard by spectrophotometry at 520 nm wavelength, to achieve a concentration of approximately 1 × 10^6^ CFU/mL (according SCLI M27-A3). It was then diluted 1:100 with Saboraud broth, and 10 µL from this last dilution were inoculated into each well, to reach an assay concentration of 1 × 10^3^ CFU/mL.

The plates were incubated for 18–20 h at the optimal growth temperature for each organism in an Incuterm, Trade Raypa®, bacteriological culture Incubator and an ERI 180 DBO, Equitec®, refrigerated incubator. The pH of the wells was measured before and after incubation to ensure that they remained in the optimal range for each microorganism, according ATCC specifications (see Support Information 1). All processes relating to the culturing, handling and inoculation of bacterial strains and the preparation of reagents were carried out under sterile conditions in a class II laminar flow biological safety hood (Model MSC Advantage 1.2).

#### Determination of antimicrobial properties

After incubation, the MIC was considered as the lowest concentration that inhibited visible microbial growth according to SCLI, M07-A9 2018^59^. To achieve a more accurate quantification of the growth, the optical density of each well was also measured at 625 nm with a BioTek™ Synergy H1 Hybrid Multi-Mode Microplate Reader.

The minimum bactericidal concentration (MBC) and minimum fungicidal concentration (MFC) were evaluated^60,61^ considering that both values indicated the lowest concentration at which all microorganisms (bacteria and fungus, respectively) were killed. For their determination, an aliquot of 10 µL from each column in which there was no growth of the incubated 96-well plates was taken and inoculated onto an agar plate. The cultures were incubated for 24 h at the optimal growth temperature for each bacterial strain (see Support Information 1) and observed to see whether any growth had occurred.

The MBC/MIC (Minimum Bactericidal Concentration/Minimum Inhibitory Concentration) ratio determines the bactericidal or bacteriostatic effect of the product on the strains. In concordance with Adrar et al.^62^, a natural product was considered to have bactericidal activity when MBC/MIC ≤ 4.

The values from the inhibition curve obtained from the micro-broth dilution test were used to calculate the LC_50_ and LC_10_ values (the concentration that causes the death of 50% or 10%, respectively, of a group of tested organisms).

### Water samples

Water samples were collected from the Gállego river (Zaragoza, Spain) and transported to the laboratory according to standard procedures. Microorganisms were extracted from 5 L of the river water for genetic analysis^49^. To do this, the sample was filtered through a 0.22 µm cellulose nitrate filter (Sartorius) with a side-arm flask under reduced pressure, resuspended in a sterile Falcon tube with 50 mL of the phosphate-buffered saline (PBS) and centrifuged for 10 min at 5000×*g*. The supernatant was discarded and the pellet was stored at − 80 °C until sequencing. For the ecotoxicity assays, 1 L of river water was filtered with a 70 µm filter of nylon (BD Falcon) to remove debris and stored at 4 °C in the dark prior to use. The physico-chemical properties of a sample of river water were also analysed (Support Information 2).

### Soil samples

The soil was obtained from a crop field, free of pesticides or other contaminants (CITA, Zaragoza, NE Spain). The soil composition was analysed by the CITA Soil and Irrigation Unit (Support Information 2). The soil microorganisms were obtained for genetic study as follows^63^: 100 mL of sterile water was added to 20 g of soil, the mixture was stirred in sterile conditions for 30 min, and the sample was then left to stand for 1 h. Then, 10 mL of the sample was divided into Falcon tubes, which were sonicated for 1 min and centrifuged at 1000×*g* for 10 min. The supernatant was collected sterile and then filtered using a 0.22 µm cellulose nitrate filter (Sartorius) with a side-arm flask under reduced pressure, and the filter containing the soil microorganisms was carefully washed with sterilized PBS and centrifuged at 5000×*g* for 10 min. The supernatant was removed with an eyedropper, and the pellets were stored at − 80 °C until sequencing.

Microorganisms for the ecotoxicity assays were obtained from 10 g of soil, which was sieved with a 2 mm sieve (Becton Dickinson, Spain), and 95 mL of sterile water, and the sample was left stirring in a flask for 30 min before standing for 1 h. Then, 10 mL from the top of the flask were placed in Falcon tubes and centrifuged for 10 min at 1000 g, with the supernatant being collected under sterile conditions. This process was repeated five times. Before obtaining the sample for inoculation of the Biolog plates, the total supernatant was filtered to remove suspended soil debris using a 70 µm sieve of nylon (Becton Dickinson, Spain).

### Community-level physiological profiling (CLPP) of river and soil microorganisms

In order to assess the effects of *S. montana* hydrolate on the metabolism of microbial communities and the changes in the use of 31 different carbon sources in water and soil, the Biolog EcoPlate test (Tiselab S.L., Spain) was used, as described previously^48^. The assay was carried out under sterile conditions in a class II laminar flow biological safety hood (Model MSC Advantage 1.2). For the Biolog tests, 0.5, 12.5, 25, 37.5 and 50% v/v dilutions of *S. montana* hydrolate were prepared in a final volume of 150 µL in the wells of a Biolog plate using prefiltered river water (see Sect. 4.5) or the supernatant obtained from the soil sample (see Sect. 4.6). Each concentration was tested in triplicate. The final pH of dilutions was between 6 and 7. The Biolog plates were incubated in the dark at 25 °C for 144 h under sterile conditions.

The OD (wavelength 590 nm) of each well was measured just after inoculation and once a day for the following 144 h, using a Synergy H1 Microplate reader (BIO-TEK. EEUU) and Gen5™ data analysis software. The microbial activity of each Biolog microplate was expressed as the average well colour development (AWCD) and determined according to Garland and Mills^64^, as in previous studies^49^:

1 $$AWCD = \sum\limits_{i = 0}^{i = 12} {\left( {OD_{{t = x_{i} }} - OD_{{t = x_{0} }} } \right)}$$

where OD_*t*=*xi*_ is the optical density value from each well at any given time and OD_*t* = *X0*_ is the optical density value from each well at the beginning of the experiment.

Among the 31 substrates, those with similar patterns of utilization were aggregated into five functional classes: amines/amides, amino acids, ketonic and carboxylic acids, polymers, and carbohydrates^65,66^, and the changes in metabolic activity were calculated for each group of metabolites, subtracting the OD values from the control.

### Representation and statistics

XLSTAT software Addinsoft (2021) was used to obtain the corresponding LC_50_ values and standard errors (SE). Dose response models were statistically tested and represented using a chi-squared test. The same software was used to calculate the variance relationship between replicates and the Student’s t-tests on two independent samples to assess significance.

### Genetic sequencing of river and soil microorganisms

Genetic sequencing was necessary to know the taxonomic composition and the predominant taxa of the river and soil samples, and thus better interpret the effect of the hydrolate on the metabolism of the microbial communities.

Genetic sequencing of water and soil microorganisms was carried out using an Illumina MiSeq Instrument under a 2 × 300 protocol in the Genomics Unit Cantoblanco, Science Park (Madrid, Spain), as described previously^51,67,68^. Briefly, the bacterial genomic DNA of samples was extracted from 200 µL aliquots after proteinase K and RNAse digestion using G-spin columns (INTRON Biotechnology, South Korea). Quant-IT PicoGreen reagent (Thermo Fischer) was used to determine the DNA concentration, and DNA samples were used to amplify the V3-V4 region of the 16S ribosomal RNA (rRNA) gene. A Bioanalyzer 2100 (Agilent, EEUU) was used to analyse individual amplicon libraries, and the concentration was estimated by real-time PCR (Kapa Biosystems). Reads were quality filtered according to Illumina standard values, demultiplexed, and fastq files were mapped against the GreenGenes database using current applications of Base Space (16S Metagenomics, Illumina). In the run, 100% of a total of 123,683 reads passed the quality filtering for river microorganisms and 100% of a total of 128,749 reads passed for soil microorganisms.

## Results

### Effects of *Satureja montana* hydrolate and carvacrol on bacteria and fungus of clinical interest

As can be seen in Table 1, the hydrolate is generally effective against a wide range of bacteria in a 50% v/v concentration. The lowest MICs were 25% v/v, for *A. baumannii, S. agalactiae* and *P. aerogenes.* For some bacteria, such as *E. coli, P. aeruginosa, K. aerogenes, L. monocytogenes* and *E. faecalis*, the hydrolate did not provoke an effect at the tested concentrations.

**Table 1:** Antimicrobial activity of *Satureja montana* hydrolate and carvacrol against all tested strains.

Bacteria/yeast	*Satureja montana* hydrolate* (% v/v)					Carvacrol (mg/mL)		
	MIC	MBC	MBC/ MIC	LC_50_ (CI 95%)	LC_10_ (CI 95%)	MIC	MBC	MBC/ MIC
*Escherichia coli* (ATCC 25922)	> 50	> 50	–	69.75 (51.90–105.55)	9.85 (7.22–12.53)	0.5	0.5	1
*Salmonella typhimurium* (ATCC 13311)	50	50	1	23.54 (21.38–25.99)	11.31 (9.43–12.97)	0.125	0.125	1
*Klebsiella pneumoniae* (C6 )	50	50	1	26.80 (22.95–32.00)	6.02 (4.62–7.39)	0.25	0.25	1
*Serratia marcescens* (ATCC 13880)	50	> 50	> 1	28.39 (26.53–30.57)	19.11 (16.79–20.90)	0.5	0.5	1
*Proteus mirabilis* (ATCC 35659)	50	> 50	> 1	22.71 (20.68–24.99)	11.29 (9.48–12.89)	–	–	–
*Pseudomona aeruginosa* (ATCC 27853)	> 50	> 50	–	44.18 (36.43–56.82)	9.53 (7.08–11.85)	> 2	> 2	–
*Klebsiella aerogenes* (ATCC 13048)	> 50	> 50	–	41.33 (36.80–47.53)	17.57 (14.22–20.44)	0.25	0.25	1
*Acinetobacter baumannii* (ATCC 19606)	25	25	1	12.87 (11.38–14.60)	3.90 (3.12–4.66)	1	1	1
*Listeria monocytogenes* (ATCC 7644)	> 50	> 50	–	211.26 (110.64–723.77)	19.71 (13.71–27.82)	0.25	0.25	1
*Pasteurella aerogenes* (ATCC 27883)	25	25	1	21.93 (20.37–23.51)	14.53 (12.50 –16.12)	0.25	0.25	1
*Bacillus subtilis* (ATCC 6633)	50	50	1	23.79 (22.08–25.61)	15.09 (12.95–16.78)	2	2	1
*Staphylococcus aureus* (ATCC 9144)	50	50	1	28.06 (26.28–30.17)	19.20 (16.89–20.95)	0.25	0.25	1
*Enterococcus faecalis* (ATCC 19433)	> 50	> 50	–	–	–	1	1	1
*Streptococcus agalactiae* (ATCC 12386)	25	25	1	19.20 (17.75–20.57)	14.42 (12.63–15.84)	1	1	1
*Candida albicans* (ATCC 10231)	50	50	1	15.22 (13.75–16.87)	6.66 (5.53–7.69)	0.8	0.8	1

CI (confidence interval), MIC (minimum inhibitory concentration), MBC (minimum bactericidal concentration).

–: test not done due to incompatibility with solvents or insufficient data.

* Main components concentration: Thymol 9.94 µg/mL, Carvacrol 243.20 µg/mL.

Carvacrol is very effective against most bacteria; for example, against *S. typhimurium* (MIC = 0.125 mg/L). For all other micro-organisms, carvacrol showed MICs ranging from 0.25 mg/mL to 2 mg/mL. The only exception was *P. aeruginosa,* against which carvacrol showed no effect at the concentrations tested. For *P. mirabilis,* no result is offered as all the tested solvents were toxic to it, across the whole concentration range.

MBC/MIC values for both the hydrolate and carvacrol were equal to 1 (when calculations were possible), showing the microbicidal effect of both products on all bacteria and on *C. albicans*. This behaviour was not observed for *S. marcescens* and *P. mirabilis* when the hydrolate was tested and for *P. aeruginosa* when carvacrol was analysed.

The standard deviation of the optical density between replicates ranged between 4.1 × 10^–3^ and 8.4 × 10^–2^ for the bacteria and fungi assayed. *A. baumannii*, and *S. aureus* showed a slightly more irregular growth; therefore, the standard deviation of their replicates was higher, up to 0.2.

Finally, Table 1 shows the LC_50_ and LC_10_ values (dose that produces death on 50 and 10% of the sample respectively) of the hydrolate in all microorganisms tested. Bacteria that have a MIC of 50% (v/v) of the hydrolate showed LC_50_ values in the range of 22.71% v/v (20.68–24.99), for *P. mirabilis,* to 28.39% v/v (26.53–30.57), for *S. marcescens,* or 28.06% v/v (26.28–30.17), for *S. aureus*. Bacteria with MICs > 50% displayed the highest LC_50_ values; for example, *L. monocytogenes*, followed by *E. coli, P. aeruginosa and K. aerogenes*. *A. baumannii* had the lowest LC_50_ value at 12.87% v/v (11.38–14.60), followed by *S. agalactiae* and *P. aerogenes*. The fungus *C. albicans* also provided a low LC_50_ value of 15.22% v/v (13.75–16.87). The LC_50_ value of *E. faecalis* is not reported, because it was reached at doses out of the range that could be used.

### Effects of *S. montana* hydrolate on fluvial community microorganisms

#### Impact on global microbial activity

**Figure 1:**
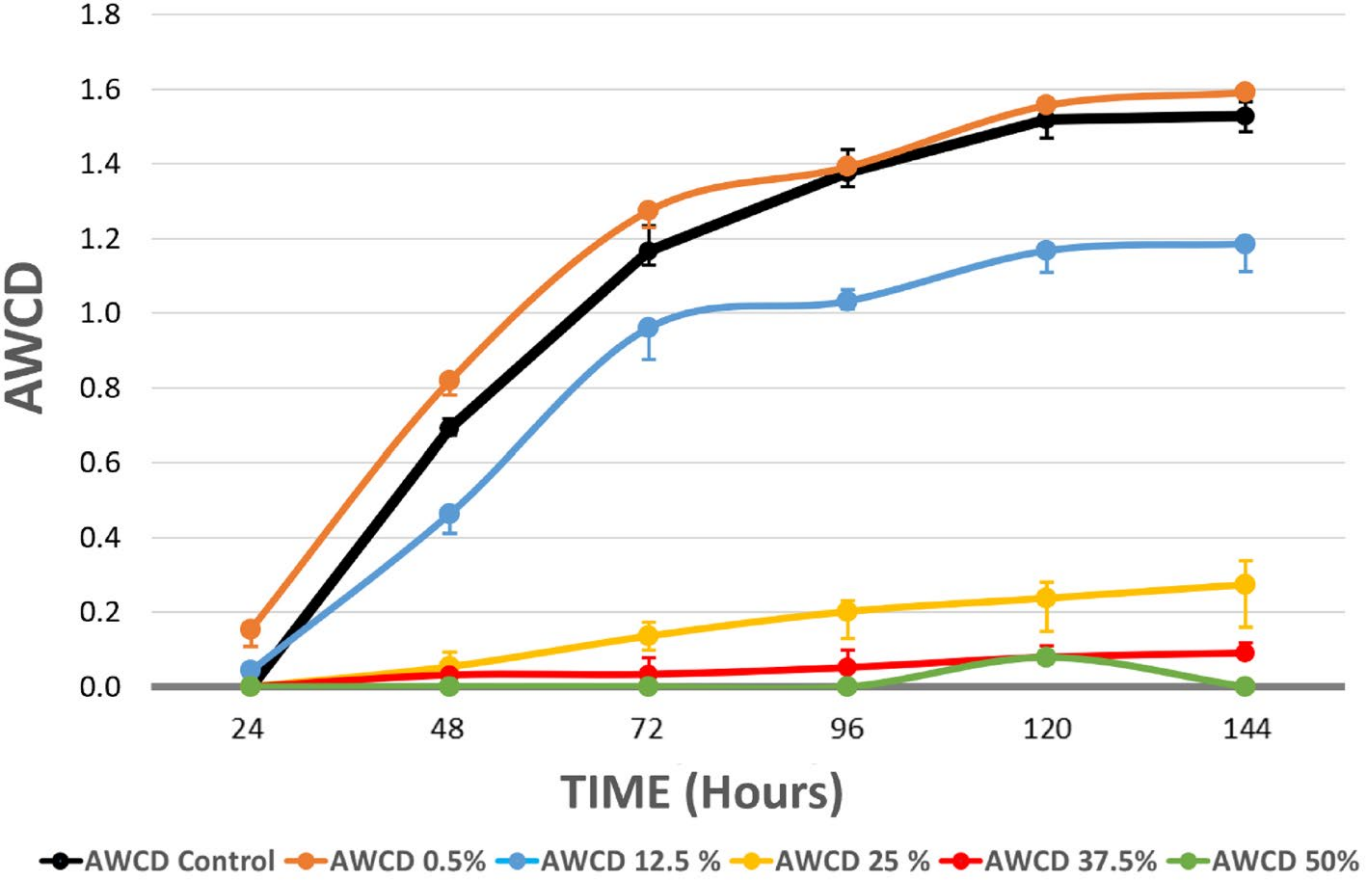
AWCD vs time curves of metabolized substrates in Biolog EcoPlates based on 144-h incubation of river microorganisms exposed to *Satureja montana* hydrolate. Black line is the negative control (microorganisms of water that have not been treated with hydrolate). Each point is the average value of three replicates. Error bars represent the standard deviation of mean of three replicates (n = 3).

The curves of the microbial activity (measured as AWCD) can be seen in Fig. 1 for the Biolog plates seeded with fluvial microorganisms and exposed to different concentrations of the *S. montana* hydrolate for 144 h.

An intense decrease in microbial activity with respect to the control (black curve) was observed in fluvial microorganisms exposed to dilutions of 25% v/v (p = 0.02) and higher concentrations (37.5 and 50% v/v; p = 0.002) of *S. montana* hydrolate. However, the decrease was already noticeable at dilutions of 12.5% (p = 0.4).

At the highest dilution (0.5% v/v), a slight empowerment in microbial activity seemed to be detected, which disappeared after 72 h. However, this increase had a low significance (p = 0.86) and was within the error range.

The AWCD values were calculated as a dose–response curve to estimate the LC_50_. At 48 h, the LC_50_ value for the hydrolate was 16.99% v/v (5.63–18.31), and the LC_10_ value was 10.19% v/v (8.73–11.45). At 72 h, the LC_50_ value for the hydrolate was 16.57% v/v (15.12–17.96), and the LC_10_ value was 9.43% v/v (7.90–10.76) (Chi-square test, p < 0.0001).

#### Changes in the ability to metabolize substrates

In addition to analysing the global impact of the hydrolate on the microbial activity of river microorganisms, the study with Biolog Ecoplates™ allows us to calculate how the metabolic profile of microorganisms is modified for each of the five functional classes: amines/amides, amino acids, ketonic and carboxylic acids, carbohydrates, and polymers.

**Figure 2:**
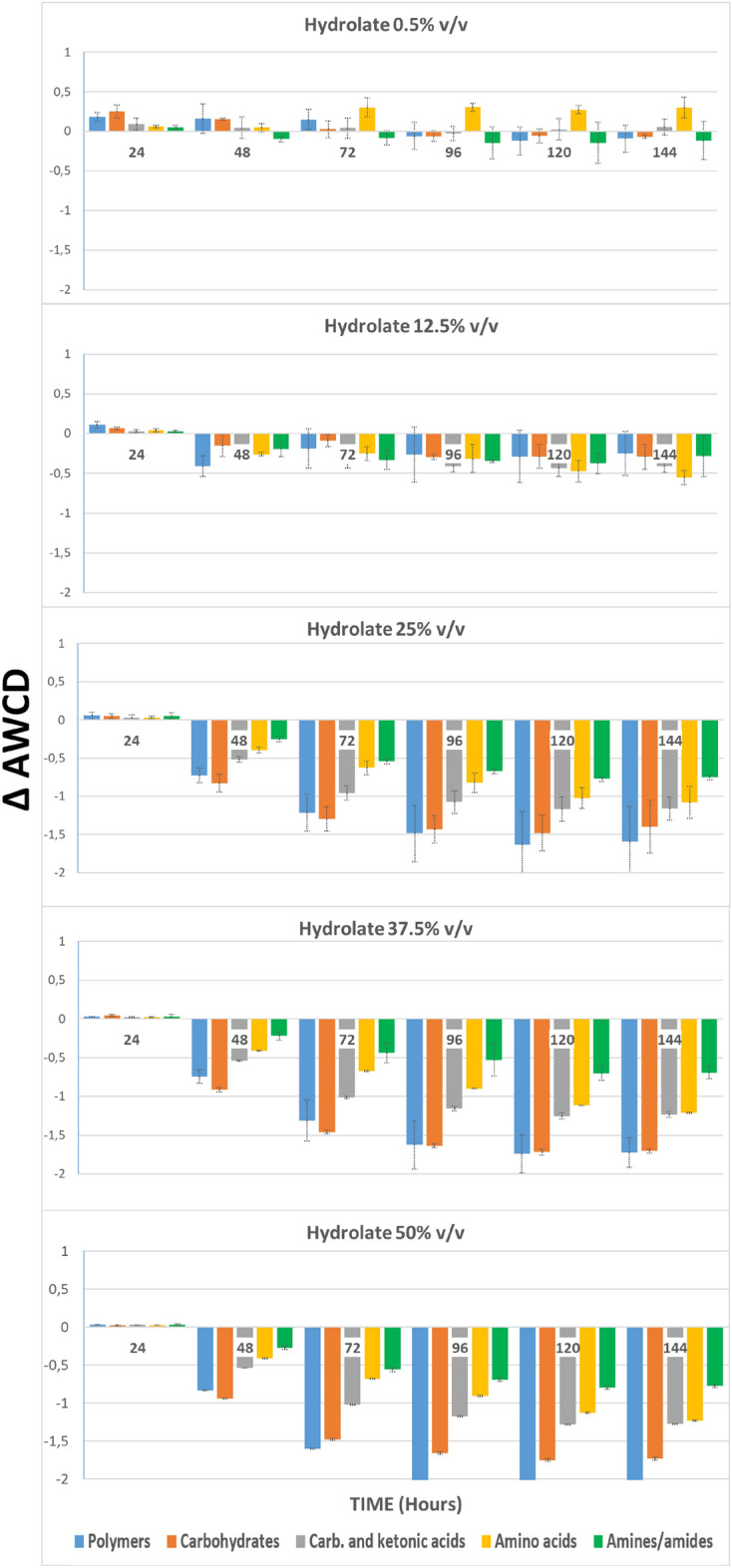
Bars represent the variation of AWCD for each group of metabolites, of water bacteria communities treated with *Satureja montana* hydrolate during 144 h. Values were obtained by subtracting those from the negative control.

In Fig. 2, ∆AWCD values (obtained by subtracting the AWCD of the negative control from the AWCD of wells with testing substance) are given. There, the changes in the capacity of the fluvial microorganisms to metabolize these five functional classes can be observed with respect to the control (x-axis), after being exposed to different dilutions of *S. montana* hydrolate for 144 h.

At low concentrations of hydrolate, (dilution 0.5% and 12,5% v/v) virtually no changes are detected.

At the 25% dilution, a drop in the ability to metabolize all metabolites was observed after 48 h and this decrease became greater as the hydrolate concentration increased (p < 0.0001). This reduction was especially intense in the case of polymers and carbohydrates.

#### Genetic sequencing of river microbial communities

**Figure 3:**
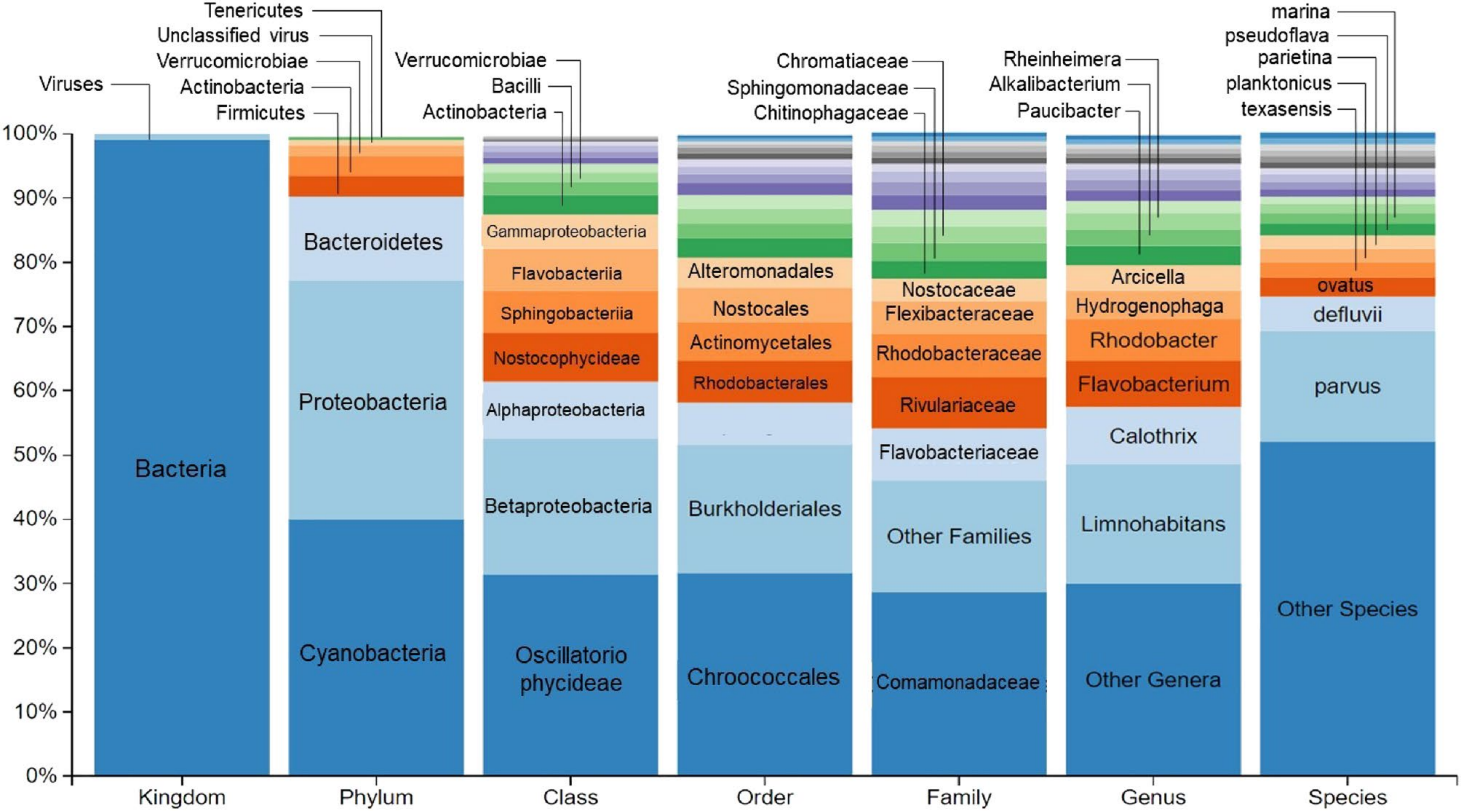
Relative abundance of microorganisms present in the river sample within each taxonomic level. From left to right: kingdom, phylum, class, order, family, genus and specie.

In Fig. 3, the taxonomic study of the bacteria in the water samples taken from the Gállego river can be observed. The 12 most abundant taxa within each taxonomic level are indicated in the figure.

There are three predominant phyla: Cyanobacteria (39.95% of reads classified to phylum level; 39.27% of total reads); Proteobacteria (37.16% of phylum level reads; 36.53% of total reads) and Bacteroidetes (13.11% of phylum level reads; 12.89% of total reads). A total of 10.33% of bacteria reads were unknown. The most abundant taxa within the Class, Order and Family can be seen in the figure. Some genera have been identified within each group such as *Calothrix* genus among Cyanobacteria or *Limnohabitans* belonging to Betaproteobacteria. *Rhodobacter* was the most abundant genus among the Alphaproteobacteria and *Flavobacterium* among Bacteroidetes.

### Effects of *S. montana* hydrolate on communities of soil microorganisms

#### Impact on global microbial activity

**Figure 4:**
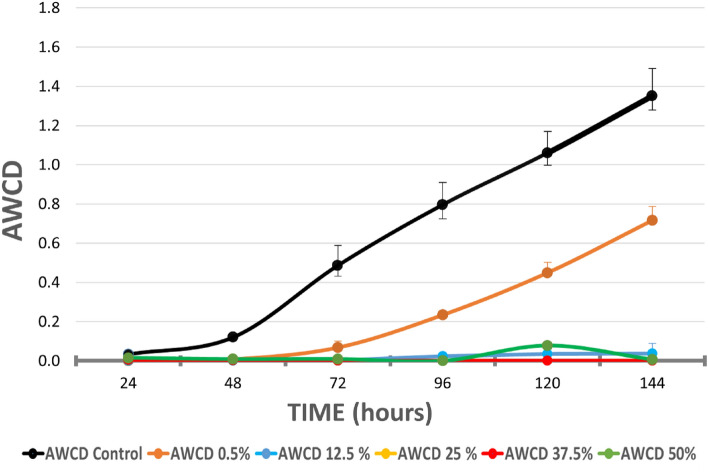
AWCD vs time curves of metabolized substrates in Biolog EcoPlates based on 144-h incubation of soil microorganisms exposed to *Satureja montana* hydrolate. Black line is the negative control (soil microorganisms that have not been treated with hydrolate). Each point is the average value of three replicates. Error bars represent the standard deviation of mean of three replicates (n = 3).

Figure 4 shows the effect of the hydrolate on the microbial activity (AWCD values) of the microorganisms obtained from the soil throughout the 144 h of the assay.

As can be seen, even at the most dilute concentration (0.5% v/v), the decrease in microbial activity with respect to the control was very evident from the beginning of the test. All of the next highest hydrolate concentrations caused almost complete inhibition of microbial activity (p < 0.05).

The LC_50_ value at 48 h was 0.078% v/v (0.012–0.181) and the LC_10_ value was 0.007% v/v (0.012–0.181), Chi-square test, p < 0.0001. The LC_50_ value at 72 h was 0.168% v/v (0.065–0.317) and LC_10_ value was 0.010% v/v (0.002–0.031), Chi-square test, p < 0.0001.

#### Changes in the ability to metabolize substrates

**Figure 5:**
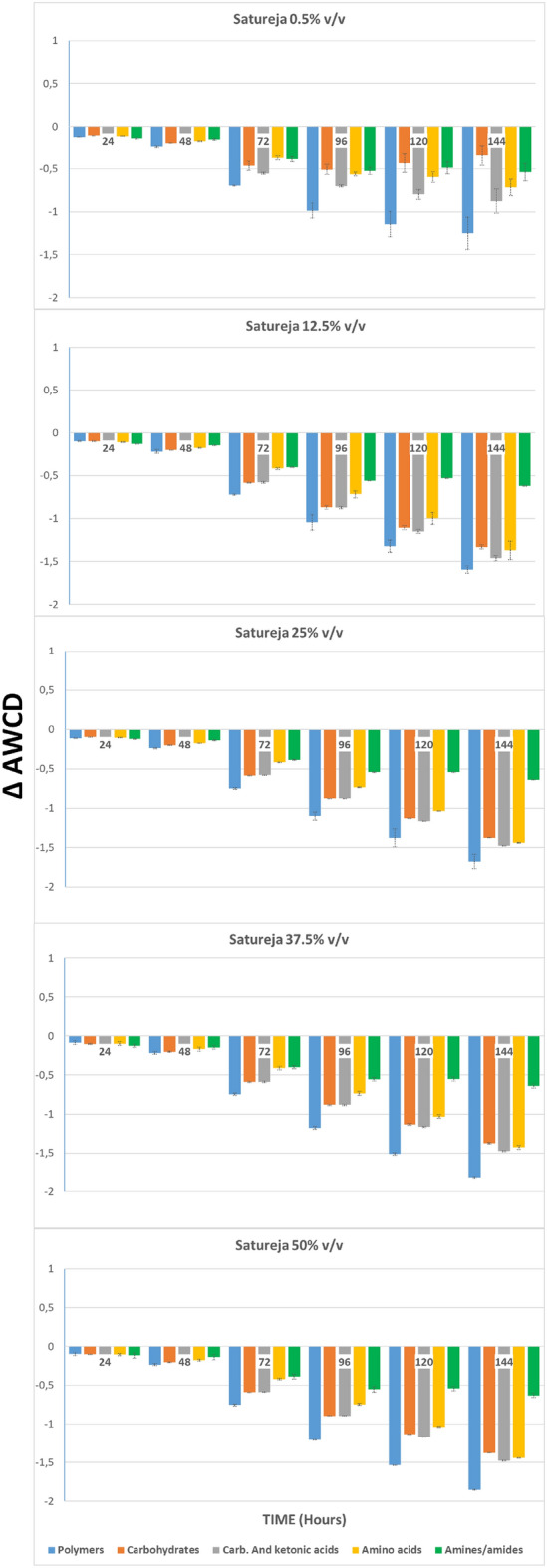
Bars represent the variation of AWCD for each group of metabolites, of soil bacteria communities treated with *Satureja montana* hydrolate during 144 h. Values were obtained by subtracting those from the negative control.

When the effect of the hydrolate on the capacity of soil microorganisms to metabolize the five functional groups of the Biolog substrates was analysed (Fig. 5), at the concentration of 0.5% v/v, a clear decrease in ∆AWCD was detected on all functional groups, especially polymers, and carboxylic and ketone acids. When soil microorganisms were exposed to higher concentrations of hydrolate, this effect increased for all functional groups, especially for polymers, and carboxylic and ketone acids, but also for amino acids.

#### Genetic sequencing of soil microorganism communities

**Figure 6:**
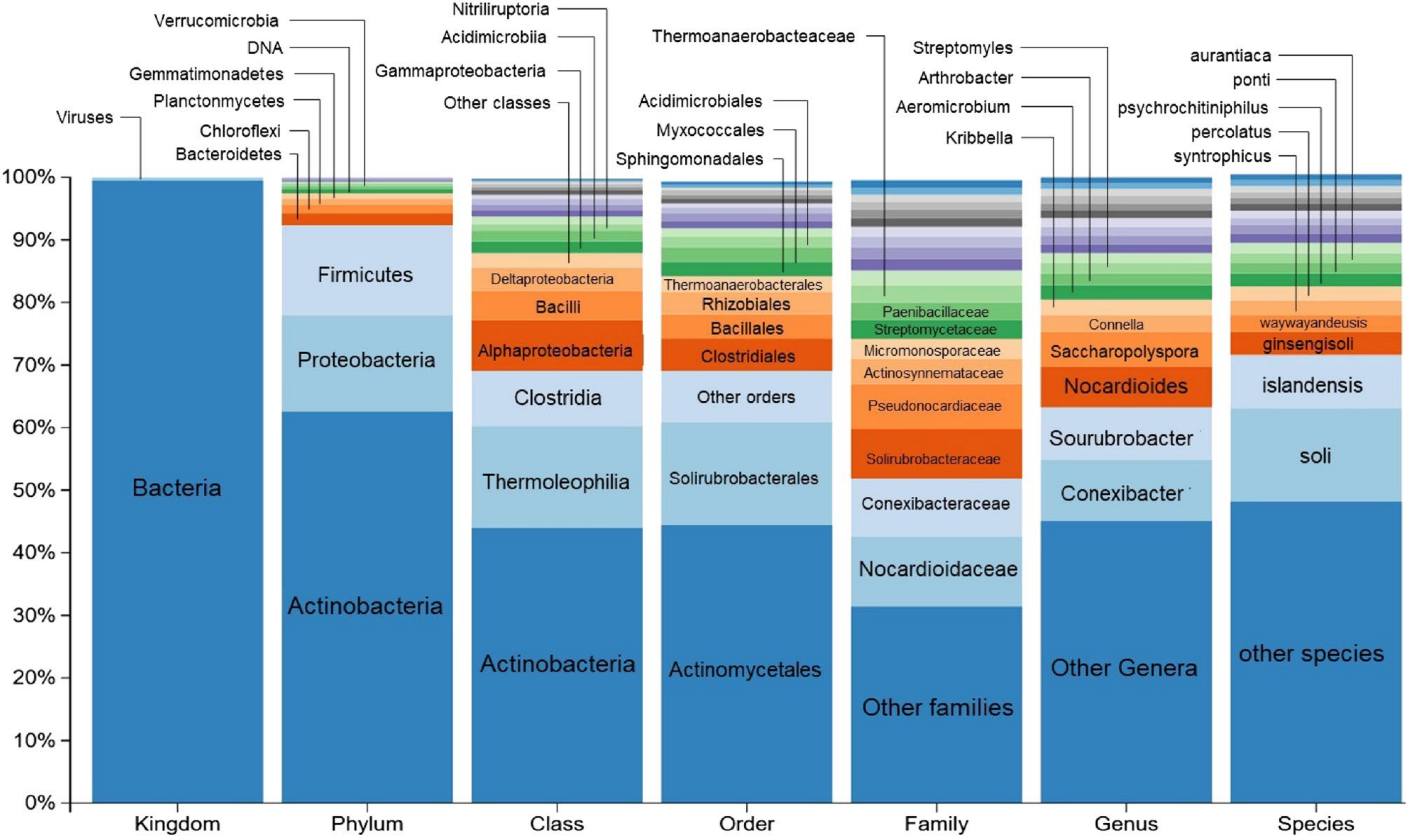
Relative abundance of microorganisms in the soil sample within each taxonomic level. From left to right: kingdom, phylum, class, order, family, genus and specie.

Figure 6 shows the taxonomic study of soil organisms organised in different taxonomic levels. Three main phyla could be seen: Actinobacteria, which was the most abundant (58.92% of bacteria reads), and Proteobacteria and Firmicutes in a similar ratio (14.50% and 13.66% of bacteria reads, respectively). A total of 12.92% of bacteria reads were unknown.

Among the phylum Actinobacteria, the most abundant class were Actinobacteria and the identified bacteria being mainly from the families Pseudonocardiacea (with the main genus, *Shaccaropolyspora*,) and Nocardioidaceae (main genus *Nocardiodes*).

The most abundant class of Proteobacteria was Alphaproteobacteria, followed by Deltoproteobacteria and Gammaproteobacteria. The most frequent orders of Alphaproteobacteria were Rhizobiales. Numerous families were found to be Rhizobiales, but practically all Sphingomonadales members belonged to the Sphingomonadaceae family, with the *Kaistobacter* genus being predominant. The order of Myxococcales was the most abundant of the Deltoproteobacteria and Xhantomonadales was the most abundant order of Gammaproteobacteria of the phylum Firmicutes, 59.20% were of the Clostridia class and 31.05% were Bacilli.

## Discussion

Our results reflect that the hydrolate of the specie *S. montana* has bactericidal properties in a wide variety of Gram-positive and Gram-negative pathogenic bacteria and a fungicidal effect on *C. albicans.*

Particularly, concentrations around 25% dilution of *S. montana* hydrolate were bioactive, reflecting the antimicrobial capacity of the hydrolate compounds, despite being present at low concentration. The hydrolate is especially bactericidal against *S. agalactiae*, *P. aerogenes* and A. *baumannii* (25% dilution of hydrolate) (Table 1). In addition, at a dilution of 50%, *S. montana* hydrolate is bactericidal against S. *typhimurium*, *Klebsiella pneumoniae*, *Bacillus subtilis* and *S. aureus* and can inhibit the growth of *S. marcescens* and *P. mirabilis*. At this concentration, it is also fungicidal for *C. albicans*. These percentages are similar to those found for other hydrolates that are considered to have an antimicrobial effect^9,25^.

To our knowledge, only Di Vito et al.^26^ have studied the effect of *S. montana* hydrolate against various pathogen microorganisms, which do not correspond to the microorganisms in our study. Although two of them are *E. faecalis* and *C. albicans*, they are not reference strains like ours and the first was also antibiotic resistant. However, the MIC values were in the same range as ours (MICs > 50% v/v for *E. faecalis* and MIC = 50% v/v for *C. albicans*). They also tested methicillin-resistant and methicillin-susceptible strains of *S. aureus*, and both appeared more resistant than the strain we tested in this study (*S. aureus* ATCC 9144). Hydrolates from other genus of Satureja also showed bactericidal activity. The hydrolate of *S. hortensis* L proved to have a bactericidal effect on *E. coli* of the same strain^21^ and on different strains^10^. This hydrolate also demonstrated bactericidal properties on *S. typhimurium*, *S. aureus* and *K. pneumonia* of different strains from ours^21,22^. However, these studies did not obtain MICs, since they used disc diffusion tests or a decrease in microbial count (cfu/mL) in freshly cut tomatoes, cucumbers or seeds. The hydrolate of *S. thymbra* was also bactericidal against the bacterial biofilms of *S. enterica* and *L. monocytogenes,* studied as a reduction of cfu/mL^23^. On the other hand, we found that the hydrolate of *S. hortensis* showed fungicidal activity in *Alternaria mali* and *Botrytis cinerea*^25^.

In addition to the MIC values, to better study the behaviour of the hydrolate, the dose–effect relationship was calculated by obtaining the LC_50_, which also allowed comparison of the effect of this hydrolate with the microbial communities of water and soil (see Table 1). As expected, bacteria with the highest MICs, such as *L. monocytogenes*, followed by *E. coli*, presented the highest LC_50_ values and *A. baumannii* presented the lowest, with an LC_50_ of 12.87% v/v (11.38- 14.60), together with *S. agalactiae*, *P. aerogenes* and the fungus *C. albicans.* Literature reported LC_50_ values for *C. albicans* are only slightly higher than ours, at 25.29% v/v^26^. The dose–effect curves (not shown) had different behaviours, which is reflected in the fact that the highest values of LC_50_ do not always coincide with the highest values of LC_10_ (see *E. coli* vs *S. marcescens*), which probably implies different mechanisms of action of the hydrolate on different microorganisms.

There is a relationship between antimicrobial activity and the most abundant compounds present in many EOs tested^69^, so the same should occur with hydrolates. When the composition of our hydrolate was analysed, the main volatile components were carvacrol and thymol (Pino-Otín et al., 2022). Although some differences in hydrolate composition can be found, even coming from the same genus, hydrolates of the genus *Satureja* have shown carvacrol as one of the predominant volatile compounds^26,70^. The antimicrobial activities of other species of *Satureja*, such as *S. hortensis* have been clearly related to the predominant presence of carvacrol and thymol in the hydrolate^10,69^. It should be noted that the hydrolate of *S. montana,* which is rich in these two compounds, had a greater antimicrobial effect than the essential oil of the same plant^26^. It is well known that carvacrol and thymol both have wide pharmacological actions^71–73^ and excellent antimicrobial properties on a wide variety of Gram-positive and Gram-negative pathogenic bacteria^74,75^*.* Our results reflect the following sensitivity to carvacrol, from the highest to the lowest: *S. typhimurium* > *K. pneumoniae* = *K. aerogenes* = *L. monocytogenes* = *S aureus* = *P. aerogenes* > *E. coli* = *S. marcescens* > *C. albicans* > *S. agalactiae* = *A. baumannii* = *E. faecalis* > *B. subtilis* > *P. aeruginosa* (see Table 1).

Among Enterobacteriaceae, the literature reports values for carvacrol on *E. coli* similar to ours of 0.4 mg/mL^76,77^. However, different values can be found, from 0.2 mg/mL^29,78^ to 0.045 mg/mL^28^. In the case of *S. typhimurium*, the MIC values for carvacrol are also highly variable: Miladi et al.^79^ and Trevisan et al.^80^ reported similar results to ours (MIC = 0.128 mg/mL and 0.312 mg/mL, respectively), but higher values of 0.4 or 0.5 mg/mL^79,81^ and values even lower than 0.064 mg/mL^79^ are also described. Literature for *K. pneumoniae* shows a wide range of MIC values for carvacrol: 0.8 mg/mL^76^ and 0.125 to 0.250 mg/mL depending on the strain^82^; this last value equals the MIC obtained in this work (Table 1). Other Gram-negative bacteria*,* such as *P. aeruginosa*, also show a wide diversity of results, from 0.25 mg/mL^82^ to 0.45 mg/mL^83^ and 0.80 mg/mL^76^. In our study, however, we did not observe effects below 2 mg/mL.

Among the Gram-positive bacteria, our MIC values for *L. monocytogenes* coincide with those reported in the literature^84^. When applying this comparison to *S. aureus,* similar carvacrol MIC values can be found, at around 0.2 mg/mL^29^, but also lower values of 0.135 mg/mL^28^ and 0.175 mg/mL^85^, and higher values of 0.482 mg/mL^83^. For *E. faecalis*, the MIC found for carvacrol (0.8 to 0.75 mg/mL)^76,86^ was similar to our results (1 mg/mL). For *B. subtilis*, most of the antimicrobial activity studies were carried out with the EO, which contained these products in different proportions, and the only MIC reported was 0.2 mg/mL for carvacrol^76^. There are no published carvacrol MICs for *S. marcescens, K. aerogenes, A. baumannii, P. aerogenes* and *S. agalactiae*, to our knowledge, although plant extracts containing carvacrol did show bactericidal activity in the first three bacteria^87–90^.

Although our values are in the range of those from other authors, we found undesired variability when comparing our results. This disparity may be a result of the different type of strain, the kind and amount of solvent used to dissolve the product, the purity and/or composition of the tested substance and even the culture medium, which are usually different in the literature^91^, and, of course, the method, among other reasons. It should be assured, for example, that the solvent used does not harm the bacteria, which is something that we have carefully considered in this study, or that the product to be tested remains dissolved throughout the entire experiment. Solubility in water is a critical aspect, since carvacrol has low solubility in water (0.11 mg/mL), which is why it is one of the parameters that limits the bioavailability of the product^92^.

There is also a large body of literature reporting the antimicrobial effects of EOs with high percentages of carvacrol or thymol on a wide variety of bacteria, many of which are present in this study^93–96^, including those of the *Satureja* genus, such as *S. bachtiarica* or *S. montana*^97,98^.

The fungicidal properties of carvacrol have been reported in the literature^30,99^ and specifically against *C. albicans*^100^. The MIC values in the bibliography are quite similar, around 0.25 mg/mL for carvacrol^101–103^, and similar to those obtained in this work, although the solvents are different (DMSO 1% and Tween-80). However, very different values (1.25 mg/mL) can also be found for carvacrol^96^. EOs rich in carvacrol and thymol also show antimicrobial activity against *C. albicans*^97,104–106^.

It should be noted that the behaviour of carvacrol alone is sometimes significantly different than that of the hydrolate on the bacteria assayed. Thus, bacteria that are most sensitive to hydrolate exposure are not necessarily the most sensitive to carvacrol (for example, *S. agalactiae*), and the most resistant to hydrolate (for example, *E. coli, K. aerogenes* and *L. monocytogenes*) are not the most resistant to carvacrol (Table 1). All this shows that, despite the fact that the antimicrobial properties of the hydrolate of *S. montana* possibly lie in the presence of carvacrol, because a clear relationship has been shown between the chemical structures of the most abundant compounds of a vegetal extract and its antimicrobial activity^21,69^, natural extracts have more complex mechanisms of action than the pure natural product. For example, the different components can establish relationships of synergy^81^, as has been described in the case of carvacrol and thymol.

Carvacrol is a hydrophobic compound, with a partition coefficient in octanol/water (P_o/w_) higher than 3. Therefore, it can penetrate deeply into the fatty acid chains of the bacteria cell membrane^107^, leading to changes in its integrity and altering its normal functioning^83^, provoking lysis. It has been suggested that the Gram-negative outer membrane may be an effective barrier against the action of these monoterpenes because it is more complex and richer in rigid lipopolysaccharides (LPS), making it more impermeable to hydrophobic compounds. However, the thick murein layer of the Gram-positive bacterial wall could not prevent the access of these antimicrobial molecules to the bacterial membrane^108,109^. Nevertheless, our results show that carvacrol affects Gram-negative bacteria as well as Gram-positive bacteria, possibly because of the other antimicrobial actions attributed to carvacrol, including the inhibition of bacteria motility, the inhibition of bacterial membrane bound ATPases and the inhibition of bacterial efflux pumps, as summarized by Kachur and Suntres^75^. The hydrolate probably also has the advantage of combining the properties of its main component with all the interactions that can be established with the components that are present in a lesser proportion.

The ability of the hydrolate and its main component to affect Gram-positive and Gram-negative bacteria may explain the results obtained for soil and water microbial communities.

Although both microbial communities are strongly affected by the hydrolate, it is clearly the soil microbial communities that show the greatest sensitivity. Concentrations of only 0.5% v/v of the hydrolate generated a decrease in bacterial growth that was very clear and already evident at 24 h. However, it was not until the exposure of dilutions of 12.5%, but especially 25%, when large effects were seen in water microorganisms. The differences in the LC_50_ values at 72 h clearly reflect this: 16.57% for the water microbial communities and 0.168% for those of soils.

In addition, the LC_50_ values of both the water and soil microbial communities exposed to the *S. montana* hydrolate are the lowest reported in the literature for other hydrolates^49–51^. This may also be partly because the microbial compositions of the river water and soil in these studies are somewhat different. Although attributing these differences to the abundance of particular taxa is speculative, some microorganisms as Chroococcales (Phylum Cyanobacteria), abundant in our water samples, frequently form colonies and gelatinous coatings and showed resistance to toxic exposure, as has been suggested in the case of metals^110^.

When the LC_50_ values of the fluvial microorganism communities are compared with the LC_50_ values of the pathogenic bacteria exposed to the hydrolate, both at 24 h, the values of the fluvial communities are almost all lower than those obtained for the pathogenic bacteria. With the exception of *A. baumannii*, (LC_50_ = 12.87%), the other microorganisms are in the range of 20%, somewhat higher for *P. aeruginosa and K. aerogenes* (slightly more than 40%), *E. coli* (LC_50_ = 69.75%) and *L. monocytogenes* (about 200%). These differences are even greater in the case of soil microbial communities, which present LC_50_ values of an order of magnitude lower (for example, 0.078% at 48 h).

This is surprising because the river or soil microbial community would be expected to be more resistant to the stresses of toxin exposure, given that there is a greater heterogeneity of organisms. This has been described, for example, in activated sludge, where the most biodiverse bacterial communities are functionally more resistant to the toxic load^111^. On the other hand, in an axenic culture, pathogenic bacteria are characterized as having a multitude of virulence factors that can make them resistant to toxic exposure. This may be the case of the bacteria that we found to be more resistant to the hydrolate as *E. coli*^112,113^, *L. monocytogenes*^114^, *P. aeruginosa*^115–118^ or *K. aerogenes*^119–121^.

The antimicrobial properties of *S. montana* hydrolate, as well as its main component, carvacrol, can certainly lead to increasing commercial applications. In fact, the hydrolate market has been growing much more than that of antibiotics in recent years (Research Report-Global Forecast 2020). Health products can now be found with carvacrol incorporated into their formulations, for example, in dental applications^122^. However, antibiotics based on carvacrol have not yet been developed.

The WHO (WHO, 2016)^32^ considers the search for new treatments against the emergence of multi-drug resistance pathogenic bacteria to be a priority, to prevent the morbidity and mortality caused by infectious agents; undoubtedly, this is going to be one of the most important challenges to human health in the twenty-first century. It should be noted that the hydrolate of *S. montana* is very effective against *A. baumannii*, one of the three bacteria among the WHO priority 1 (critical) (WHO, 2017), due to the large number of multi-resistant strains for which new antibiotics are urgently needed. Other bacteria on which the hydrolate is effective are *P. mirabilis, K. pneumoniae* and *S. marcescens* of the Enterobacteriaceae family, also in priority 1 of the WHO classification. *S. typhimurium* and *S. aureus*, on the other hand, which are also sensitive to the hydrolate, are in WHO priority 2 (high). The efficacy of the hydrolate against *C. albicans* makes it suitable for application to mucosa for the treatment of vaginal infections, for example, when EOs are contraindicated, applications that have also been suggested for other hydrolates^26^.

Hydrolates by their nature can act as antimicrobial creams and ointments on the skin, for infections that need daily local treatments and are resistant to commercial antibiotics. The increase in their use in formulations of cosmetic products for body care cannot be ruled out either.

Lastly, their joint application with commercial antibiotics can help to reduce the dose of commercial antibiotics, due to their increasingly described synergistic properties^151^.

Even though carvacrol is considered to be a safe product for use in food for human consumption, as well as being a food additive approved by the Federal Drug Administration (FDA) and included by the Council of Europe in the list of chemical flavourings that can be found in various commercial products, the effect on non-target microorganisms in the environment has not been considered until now. In fact, the environmental effect of most hydrolates that have been described as bioactive is also unknown. There are few studies that allow a comparison between the ecotoxicity of hydrolates and antibiotics under the same experimental conditions or with the same endpoints. We have had the opportunity to do similar studies with various antibiotics^123^. These results indicate, for example, that AWCD values decrease with regard to the control generated after 6 days of exposure of communities of soil microorganisms to Satureja hydrolate at concentrations of 0.5% (46.97%) would be similar to those caused by 100 µg/mL of chloramphenicol (37.79% of decrease) and gentamicin (49.10%) or 1000 µg/mL of ampicillin (38.63%), penicillin (33.59%) or streptomycin (37.23%). Other antibiotics such as erythromycin seem to have a greater impact (23% decrease in the AWCD value compared to the control at 100 µg/mL).

Therefore, the results of this study reveal that, although plant hydrolates could be a suitable alternative or a complement to commercial antibiotics, their discharge to the environment can generate changes in the structure of soil and water microbial communities; consequently, their effect on the environment must be taken into account. Furthermore, if these plant-based by-products want to be revalued to replace synthetic product and length its life cycle with useful applications, other aspects must also be considered from the perspective of One Health. For example, if they produce less resistance than synthetic antibiotics, as indicated by early studies^14^, or if they can degrade or lose activity more quickly than commercial antibiotics in the environment.

## Conclusions

The *S. montana* hydrolate exhibits antimicrobial activity against a wide range of Gram-positive and Gram-negative bacteria and against the fungus *C. albicans*. It is especially effective against: *P. aerogenes*, *S. agalactiae* and *A. baumannii*, which is considered to be priority 1 by the WHO. However, this hydrolate generates important effects on non-target organisms of the natural communities of soil and river microorganisms, made up of common groups of Cyanobacteria, Proteobacteria, Bacteroidetes, Actinobacteria and Firmicutes, and can inhibit the growth of bacteria in these communities and change their metabolic profile, with special intensity on edaphic bacteria.

The interesting antimicrobial capacity of hydrolates means that their use as antibiotics should be explored, either to replace traditional antibiotics in some cases, or to act in synergy with them to allow the use of lower doses. However, the results of this work reflect that these products also have some impact on the environment, which must be taken into consideration, as with traditional antibiotics.

## Supplementary Information

Supplementary Information.

## Data Availability

The datasets generated during and/or analysed during the current study are available from the corresponding author on reasonable request.
