# Supplementary material for: Antibiotic properties of Satureja montana L. hydrolate in bacteria and fungus of clinical interest and its impact in non-target environmental microorganisms
Source: Sci Rep. 2022 Nov 2;12:18460. doi: 10.1038/s41598-022-22419-2 (PMC9630514; doi:10.1038/s41598-022-22419-2)

**Support information 1**. Microorganisms reference, culture conditions according to ATCC datasheets, solvent and its concentration used for each microorganism.

| **Microorganism culture conditions** | | | | | | |
| --- | --- | --- | --- | --- | --- | --- |
| **Microorganism** | **Reference** | **GRAM** | **Temperature (ºC)** | **Time (h)** | **Agar/Broth** | **Used solvent (%v/v)** |
| *Escherichia coli* | ATCC 25922 | negative | 37 | 24 | TS | Ethanol 5% |
| *Salmonella typhimurium* | ATCC 13311 |  |  |  | NU |  |
| *Klebsiella pneumoniae* | C6 |  |  |  |  | DMSO 2.5% |
| *Serratia marcescens subsp.marcescens* | ATCC 13880 |  | 26 | 24-48 |  |  |
| *Proteus mirabilis* | ATCC 35659 |  | 37 | 24 | TS | - |
| *Pseudomonas aeruginosa* | ATCC 27853 |  |  |  |  | DMSO 2.5% |
| *Klebsiella aerogenes* | ATCC 13048 |  | 30 |  | NU |  |
| *Acinetobacter baumannii* | ATCC 19606 |  | 37 |  |  |  |
| *Listeria monocytogenes* | ATCC 7644 |  |  |  | BHI |  |
| *Pasteurella aerogenes* | ATCC 27883 |  |  |  |  |  |
| *Bacillus subtilis subsp.spizizenii* | ATCC 6633 | positive | 30 |  |  |  |
| *Staphylococcus aureus subsp. aureus* | ATCC 9144 |  | 37 |  | TS | TWEEN 80 1.25% |
| *Enterococcus faecalis* | ATCC 19433 |  |  |  | BHI | DMSO 2.5% |
| *Streptococcus agalactiae* | ATCC 12386 |  |  |  |  |  |
| *Candida albicans* | ATCC 10231 | - | 24-26 | 48 | SD | TWEEN 80 1.25% |
| TS -Trypticase Soy Agar/Broth, NU - Nutrient agar or nutrient broth, BHI - Brain Heart Infusion Agar/Broth, SD - Sabouraud dextrose agar/broth. DMSO - Dimethyl sulfoxide. | | | | | | |

**Support information 2**. Physico-chemical parameters of the river water sample collected from the Gállego River (Ebro river tributary; Montañana, Zaragoza, Spain) on June, 2019 for water Biologs assays. In the columns on the right, composition of Soil samples collected from a crop field free of contaminants from CITA (Zaragoza, NE-Spain) on July ,2019 for soil Biologs assays.

| **River Water parameters** | **Value** |
| --- | --- |
| Conductivity (µs/cm) | 2317 |
| pH | 7.8 |
| Total suspended solids (mg/L) | 5.1 |
| Organic materia (mg/L) | 3.0 |
| Total dissolved solids (mg/L) | 1586.4 |
| Carbonates (mg/L) | 0.0 |
| Bicarbonates (mg/L) | 249.4 |
| Fluorides (mg/L) | 0.1 |
| Chlorides (mg/L) | 475.2 |
| Nitrites (mg/L) | 0.0 |
| Bromides (mg/L) | 0.5 |
| Nitrates (mg/L) | 14.5 |
| Phosphates (mg/L) | 0.0 |
| Sulphates (mg/L) | 358.8 |
| Total alkalinity (mg/L) | 249.4 |
| Total organic carbon (mg/L) | 3.0 |
| Total nitrogen (mg/L) | 3.8 |
| **Soil parameters** | **Value** |
| Clay content (%) | 11.7 |
| Sand content (%) | 50.5 |
| Silt content (%) | 37.8 |
| pH | 8.1 |
| K (mg/L) | 109.3 |
| P Olsen (mg/Kg) | 10.4 |
| Ca (meq/100) | 23.8 |
| EC_1:5_ (dS/m) | 0.6 |
| Total nitrogen (%) | 0.1 |
| CaCO_3_ (%) | 42.3 |

**Support information 3**. Steam distillation process for the extraction of *S. montana* hydrolate. (a) Details of the process steps. Semi-industrial pilot extraction plant used for the extraction of hydrolate can be seen in: <https://www.cita-aragon.es/planta-piloto/>.


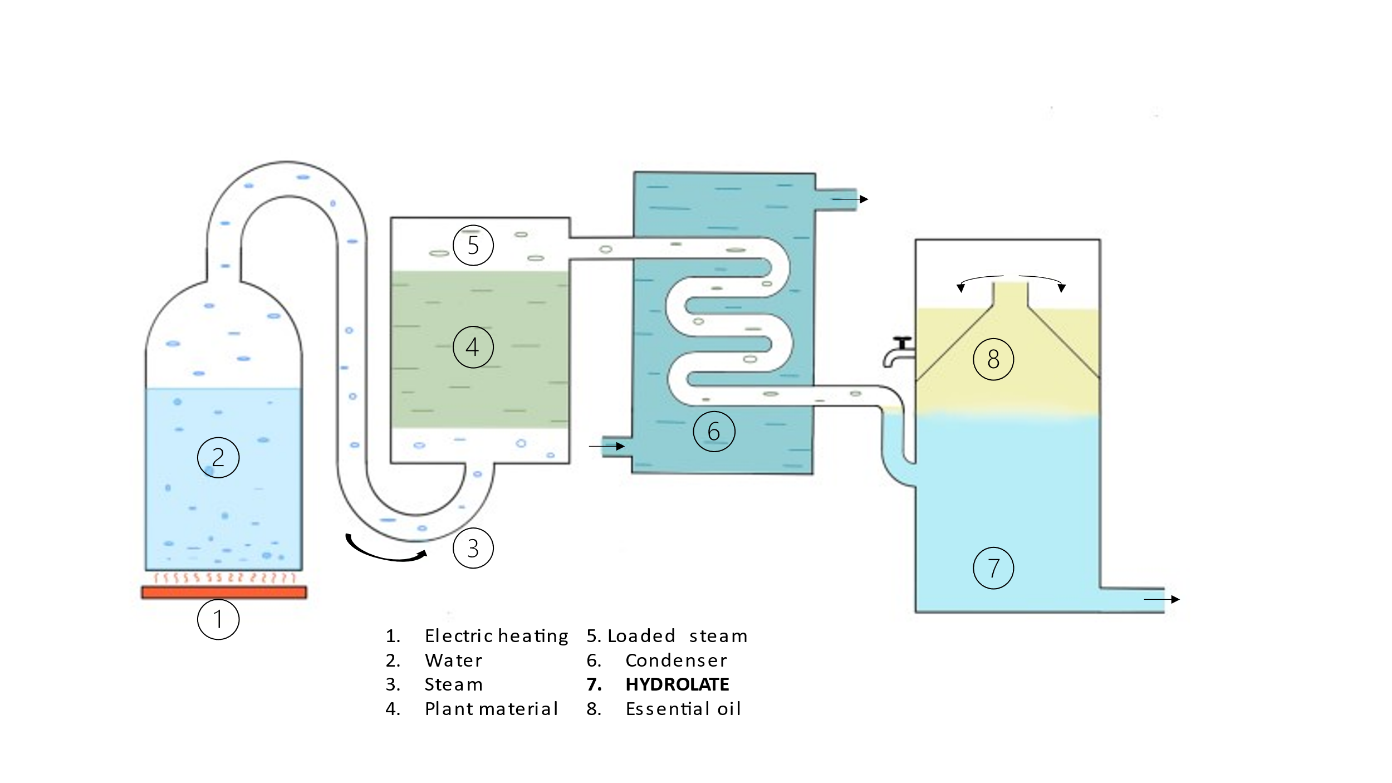

Supplement: Supplementary file 1 — Supplementary Information. [file 41598_2022_22419_MOESM1_ESM.docx]
